# Memoirs of Medicine; Including a Sketch of Medical History, from the Earliest Accounts to the Eighteenth Century

**Published:** 1799-08

**Authors:** 


### MEDICAL HISTORY.

*Memoirs of Medicine; including a Sketch of Medical History, from the earliest accounts to the Eighteenth Century.* By RICHARD WALKER, Esq. Apothecary to the Prince of Wales, *London*, 1799, (five shillings) Johnson.

The author commences this work with a general outline of the history of medicine medicine, from the earlieſt ages, prior to the era of the Greeks, and he makes ſome good remarks on the ſtate of medicine among that celebrated people. He extends his reſearch far beyond the time of Hippocrates, gives a ſhort biography of that illuſtrious character, and pays due tribute to his genius, as well as to his enlightened and liberal ſentiments. After ſeveral intereſting memoirs reſpecting the practitioners of Greece, he ſtates conciſely the diſcoveries of Eraſiſtratus and Herophilus, relative to the ſenſibility and power of the brain and nerves. His account of the origin of the medical ſect, called Methodiſts, is curious; he proceeds (in chronological order) to take a ſhort view of the different medical revolutions which have happened in different quarters of the globe, and after paying deſerved homage to his countrymen, Harvey, Sydenham, and others, he concludes the ſubject with a reference to "the preſent ſtate of medicine in England;" the account of which is extremely conciſe, as it ſcarcely fills eight pages. Although this little work poſſeſſes much merit, and deſerves to be recommended to the medical tyro, eſpecially ſince the Hiſtory of Medicine is too much neglected by a certain claſs of readers, who wiſh for nothing but '*new facts* and *improvements*;' yet we apprehend, that the ſtyle in which it is written will be deemed rather too florid by the plurality of readers. Laſtly, we ought to mention that this work is dedicated to the Prince of Wales.

